# Environmental risk factors of systemic lupus erythematosus: a case–control study

**DOI:** 10.1038/s41598-023-36901-y

**Published:** 2023-06-23

**Authors:** Rania H. Refai, Mohammed F. Hussein, Mamdouh H. Abdou, Anna N. Abou-Raya

**Affiliations:** 1 Department of Medicine Supply and Pharmacy, Alexandria University Hospitals, Alexandria University, Alexandria, Egypt; 2 Department of Occupational Health and Industrial Medicine, High Institute of Public Health, Alexandria University, Alexandria, Egypt; 3 Department of Internal Medicine, Rheumatology & Clinical Immunology, Faculty of Medicine, Alexandria University, Alexandria, Egypt

**Keywords:** Rheumatology, Risk factors

## Abstract

Systemic lupus erythematosus (SLE) is a complicated chronic autoimmune disorder. Several genetic and environmental factors were suggested to be implicated in its pathogenesis. The main objective of this study was to examine how exposure to selected environmental factors was associated with SLE risk to support the development of disease preventive strategies. A case–control study was conducted at the Rheumatology outpatient clinic of Alexandria Main University Hospital, in Alexandria, Egypt. The study sample consisted of 29 female SLE patients, and 27 healthy female controls, who matched the cases on age and parity. Data were collected by a structured interviewing questionnaire. Blood levels of lead, cadmium, and zinc of all participants were assessed by flame atomic absorption spectrometry. The multivariate stepwise logistic regression model revealed that five factors showed significant association with SLE, namely living near agricultural areas, passive smoking, blood lead levels ≥ 0.075 mg/L, and exposure to sunlight (odds ratio (OR) 58.556, 95% confidence interval (CI) 1.897–1807.759, OR 24.116, 95% CI 1.763–329.799, OR 18.981, 95% CI 1.228–293.364, OR 9.549, 95% CI 1.299–70.224, respectively). Whereas walking or doing exercise were significantly protective factors (P = 0.006). The findings of this study add to the evidence that SLE can be environmentally induced. Preventive measures should be taken to address the environmental risk factors of SLE.

## Introduction

Systemic lupus erythematosus (SLE) is a chronic rheumatic autoimmune disorder that could be manifested by many symptoms. It is a multi-system disease that may involve nearly any organ resulting in serious organ complications and even death^1,2^. It predominantly affects women in the child-bearing ages^3^. SLE was suggested to be a condition of multifactorial etiology; including genetics, hormones, and environmental exposures.

It is currently known that different environmental factors could trigger SLE onset and flares in genetically susceptible individuals^4,5^. Owing to the increasing prevalence and overall SLE burden, efforts have been made to recognize these genetic and non-genetic factors^6^.

The role of the environment was more prominent by the fact that SLE concordance among identical monozygotic twins is below 25%^7^. In addition, the contribution of environmental factors to SLE risk has been evaluated to constitute 56%^8^.

Smoking, silica dust, UV radiation, infections, stress, air pollution, pesticides, and heavy metals are the major environmental risk factors having some evidence for association with SLE^9^.

In recent years, heavy metal pollution has become a significant health issue; continuous exposure to low levels of these toxic trace elements may result in bioaccumulation and produce a wide variety of biological effects on human beings^10^.

Lead and cadmium are known to pose serious risks to human health. Toxicity of these agents is evidenced by being identified in the top 10 environmental hazards by the Agency for Toxic Substances and Disease Registry^11^.

Environmental sources of lead include inhalation of airborne dusts containing lead and ingestion through food or water contaminated by lead. Old deteriorating household paints, and lead use in some traditional medicines and cosmetics can also be a source of lead exposure^12^. In addition, active and passive smoking were found to be associated with increased blood lead levels^13^.

Cadmium is present in cigarette smoke, air, food, and water. It can enter human bodies through inhalation, ingestion and dermal contact^14^.

Experimental studies of lead and cadmium exposure in rodent models proposed that metals may play a causative role in SLE^15,16^. There is relatively little data pertaining to lead or cadmium exposure with the risk of SLE in humans; exposure to stained or leaded glass as a hobby was found to be more common among SLE cases than controls^17^.

Trace elements such as zinc play a crucial role in growth and development of all organisms. Zinc is the second most abundant trace metal in the human body after iron, but zinc cannot be stored and has to be taken up daily via food to guarantee sufficient supply. However, it was stated that zinc excess as well as zinc deficiency may result in severe disturbances in immune cell numbers and activities leading to immune dysfunction^18^.

A study by Sahebari et al.^19^ found that serum Zn values were lower in SLE patients than healthy age and sex-matched controls. On the other hand, zinc-deficient diets retarded autoantibody production and enhanced survival in mice^20^. In their review, Constantin et al.^21^ advised to restrict consumption of some minerals such as zinc and sodium.

As the data about the relation between environmental risk factors and SLE in Egypt are lacking and the incidence is increasing, the present study was proposed to determine the association between some environmental exposures with SLE risk; in order to assess the extent to which SLE is environmentally induced.

## Results

### Estimation of SLE risk in relation to some socio-demographic characteristics

Analysis of data regarding the socio-demographic characteristics revealed that patients and controls were similar in terms of demographic background, no statistical significant difference between cases and controls except for the education level, the occupation, and the residence near agricultural areas (Tables 1, 2).

**Table 1:** SLE risk estimation in relation to some sociodemographic characteristics (Rheumatology clinic, Alexandria University Hospital, 2020).

Item	Cases (n = 29) N (%)	Controls (n = 27) N (%)	Test of significance (P value)	Odds ratio OR (95% CI)
Age				
20–	9 (31)	12 (44.4)	X^2^ = 1.07 (P = 0.3)	0.563 (0.188–1.67)
35+ (Ref)	20 (69)	15 (66.6)		1
Mean ± SD	37.48 ± 8.378	37.81 ± 9.872	t = − 0.136 (P = 0.892)	
Marital status				
Married	27 (93.1)	23 (85.2)	0.414^✝^	2.34 (0.39–14.01)
Single/divorced/widow (Ref)	2 (6.9)	4 (14.8)		1
Level of education				
Illiterate	9 (31)	4 (14.8)	0.010^+^*	9 (0.75–108.31)
Primary/preparatory	11 (37.9)	3 (11.1)		14.67 (1.16–185.23)
Secondary/above intermediate	8 (27.6)	16 (59.3)		2 (0.19–20.97)
University/post graduate (Ref)	1 (3.4)	4 (14.8)		1
Work				
Not working	25 (86.2)	9 (33.3)	X^2^ = 16.388 (P = 0.001)*	12.5 (3.32–47)
Working (Ref)	4 (13.8)	18 (66.7)		1

*Ref* reference, *X*^*2*^ Chi-square, + Monte Carlo, ^✝^Fisher, *t* t-test, *P < 0.05.

**Table 2:** SLE risk estimation in relation to residence area (Rheumatology clinic, Alexandria University Hospital, 2020).

Item	Cases (n = 29) N (%)	Controls (n = 27) N (%)	Test of significance (P value)	Odds ratio OR (95% CI)
Heavy traffic				
Yes	10 (34.5)	12 (44.4)	X^2^ = 0.582 (P = 0.446)	0.66 (0.22–1.93)
No (Ref)	19 (65.5)	15 (55.6)		1
Petrol station				
Yes	0 (0)	2 (7.4)	0.228✝	0.17 (0.01–3.77)
No (Ref)	29 (100)	25 (92.6)		1
Rubbish				
Yes	6 (20.7)	2 (7.4)	0.254^✝^	3.26 (0.6–17.81)
No (Ref)	23 (79.3)	25 (92.6)		1
Factory				
Yes	5 (17.2)	2 (7.4)	0.424^✝^	2.6 (0.46–14.73)
No (Ref)	24 (82.8)	25 (92.6)		1
Agriculture				
Yes	14 (48.3)	2 (7.4)	X^2^ = 11.443 (p = 0.001)*	11.67 (2.32–58.6)
No (Ref)	15 (51.7)	25 (92.6)		1
Wireless station				
Yes	0 (0)	2 (7.4)	0.228✝	0.17 (0.01–3.77)
No (Ref)	29 (100)	25 (92.6)		1

*Ref* reference, *X*^*2*^ Chi-square, ^✝^Fisher, *t* t-test, *P < 0.05.

Regarding the educational level, there was a statistically significant difference between cases and controls (P < 0.05). The percentage of illiterate cases was (31%) and constituted more than double their percentage in the control group (14.8%). Also, the percentage of cases with higher education was only (3.4%), which is very low compared to controls (14.8%). The findings showed that females with SLE who were in the primary or preparatory educational level were significantly more likely to be at risk of SLE (OR 14.67, 95% CI 1.16–185.23) compared to controls.

With regard to occupation, there was a statistically significant difference between cases and controls (P < 0.05), the majority of cases were housewives (86.2%) compared to (33.3%) in the controls group.

### Estimation of SLE risk in relation to some lifestyle factors

Lack of physical activity, exposure to domestic animals, or to sun light showed significant results (Table 3). As regards walking and physical activity, a high proportion of SLE patients (89.7%) do not like to walk or perform any physical activity compared to (40.7%) in the control group.

**Table 3:** SLE risk estimation in relation to some lifestyle factors (Rheumatology clinic, Alexandria University Hospital, 2020).

Item	Cases (n = 29) N (%)	Controls (n = 27) N (%)	Test of Significance (P value)	Odds Ratio OR (95% CI)
Physical activity/walking				
Yes (Ref)	3 (10.3)	16 (59.3)	X^2^ = 14.923	1
No	26 (89.7)	11 (40.7)	(P = 0.001)*	12.61 (3.05–52.17)
Domestic animals raising				
Yes	17 (58.6)	8 (29.6)	X^2^ = 4.755	3.36 (1.11–10.19)
No (Ref)	12 (41.4)	19 (70.4)	(P = 0.029)*	1
Use of kohl				
Yes	9 (69)	5 (18.5)	X^2^ = 1.168	1.980 (0.567–6.909)
No (Ref)	20 (31)	22 (81.5)	(P = 0.280)	1
Use of hair dyes				
Yes	10 (34.5)	6 (22.2)	X^2^ = 1.030	1.842 (0.562–6.038)
No (Ref)	19 (65.5)	21 (77.8)	(P = 0.310)	1
Use of lipstick				
Yes	6 (20.7)	5 (18.5)	X^2^ = 0.042	1.148 (0.306–4.309)
No (Ref)	23 (79.3)	22 (81.5)	(P = 0.838)	1
Sun exposure				
Yes	24 (82.8)	7 (25.9)	X^2^ = 18.274	13.714 (3.768–49.920)
No (Ref)	5 (17.2)	20 (74.1)	(P = 0.001)*	1

*Ref* reference, *X*^*2*^ Chi-square, *P < 0.05.

Sedentary lifestyle increased SLE risk by 12.61 folds (OR 12.61, 95% CI 3.05–52.17). Dealing with domestic animals was another risk factor that was tested in the current study; the results showed that 58.6% of cases were exposed to animals more than controls (29.6%). This may be because 48.3% of cases live in rural areas compared to 7.4% of controls. There was a statistically significant increase in SLE risk when dealing with farm animals (sheep, chicken) (OR 3.36, 95% CI 1.11–10.19).

Results about UV radiation exposure and SLE risk showed a statistically significant increase of SLE risk (OR 13.714, 95% CI 3.768–49.920).

### Estimation of SLE risk in relation to some indoor environmental risk factors

The risk values for the association between some indoor environmental exposures and SLE were statistically not significant except for the type of drinking water, the filled tube cooker as the fuel used at home, and passive smoking (Table 4); 62.1% of SLE patients use tap water for drinking compared to 22.2% of controls, meanwhile, the majority of controls (74.1%) use filtered water compared to 37.9% of cases. The use of filtered water for drinking was assumed to have protective effects against SLE.

**Table 4:** SLE risk estimation in relation to some indoor environmental risk factors (Rheumatology clinic, Alexandria University Hospital, 2020).

Item		Cases (n = 29) N (%)	Controls (n = 27) N (%)	Test of significance (P value)	Odds ratio OR (95% CI)
Bad water quality					
Yes		23 (79.3)	16 (59.3)	X^**2**^ = 2.659 (P = 0.103)	2.635 (0.808–8.592)
No (Ref)		6 (20.7)	11 (40.7)		1
Drinking water pipes					
New (Ref)		18 (62.1)	16 (59.3)	X^**2**^ = 0.046 (P = 1.000)	1
Old		11 (37.9)	11 (40.7)		0.89 (0.3–2.6)
Type of drinking water					
Bottled		0 (0)	1 (3.7)	0.003^+^*	0.12 (0–3.25)
Filtered		11 (37.9)	20 (74.1)		0.18 (0.06–0.6)
Tap water (Ref)		18 (62.1)	6 (22.2)		1
Type of paint					
Wallpaper		1 (3.4)	6 (22.2)	0.152^**+**^	0.15 (0.02–1.37)
Cement		3 (10.3)	1 (3.7)		2.67 (0.25–28.28)
Old		7 (24.1)	4 (14.8)		1.56 (0.38–6.31)
New (Ref)		18 (62.1)	16 (59.3)		1
Fuel used at home					
Natural gas pipes (Ref)		6 (20.7)	20 (74.1)	X^**2**^ = 16.021 (P = 0.001)*	1
Gas cylinder		23 (79.3)	7 (25.9)		10.95 (3.16–38.01)
Passive smoking at home					
Yes		18 (62.1)	8 (29.6)	X^**2**^ = 5.916 (P = 0.015)*	3.886 (1.273–11.861)
No (Ref)		11 (37.9)	19 (70.4)		1
Degree of smoking (CPD)					
No (Ref)		11 (37.9)	19 (70.4)	0.035^+^*	1
Light		12 (41.4)	7 (25.9)	X^2^ for trend = 5.87 (0.01543)	2.96 (0.9–9.75)
Heavy		6 (20.7)	1 (3.7)		10.36 (1.1–97.69)

*Ref* reference, *X*^*2*^ Chi-square, + Monte Carlo, *CPD* the number of cigarettes smoked per day, heavy (CPD ≥ 20), light (CPD < 20), *P < 0.05.

A statistically significant difference (X^2^ = 16.021, P = 0.001) between cases and controls with regard to fuel used at home, where 79.3% of cases reported the use of gas cylinders compared to 25.9% of controls. The use of gas cylinders constituted 10.95 times more risk to SLE than the use of natural gas pipes (OR 10.95, 95% CI 3.16–38.01).

Regarding passive smoking at home, which was assessed by the number of active smokers who were residing in the same house, (62.1%) of cases were exposed to Environmental Tobacco Smoke (ETS) versus (29.6%) of controls, there was a statistically significant difference between cases and controls with regard to exposure to ETS (OR 3.886, 95% CI 1.273–11.861).

Moreover, SLE risk increased with the increase in the number of cigarettes smoked per day (the risk was 2.96 and 10.36 times for light and heavy passive smoking (OR 2.96, 95% CI 0.9–9.75; OR 10.36, 95% CI 1.1–97.69 respectively). A significant trend for risk was noticed with increased passive smoking (X^2^ for trend = 5.87, P = 0.015) concluding that ETS may be an important risk factor for SLE.

### Estimation of SLE risk in relation to a family history of any auto-immune disease

The study found a statistically significant difference between cases and controls regarding the family history of any auto immune disease as illustrated in (Table 5), 33.3% of controls had surprisingly a family history of an auto immune disease including SLE compared to 3.4% of cases.

**Table 5:** SLE risk estimation in relation to family history of any auto immune disease and hormonal factors (Rheumatology clinic, Alexandria University Hospital, 2020).

Item	Cases (n = 29) N (%)	Controls (n = 27) N (%)	Test of significance (P value)	Odds ratio OR (95% CI)
Family history of any auto immune disease				
No (Ref)	28 (96.6)	18 (66.7)	X^2 = 8.513 (0.004)*^	1
Yes	1 (3.4)	9 (33.3)		0.07 (0.01–0.61)
Menopause				
Early	9 (31)	0 (0)	0.001^+^*	31.26 (1.7–575.54)
No (Ref)	15 (51.7)	25 (92.6)		1
Menstrual cycle				
Regular (Ref)	9 (60)	23 (92)	0.036✝*	1
Irregular	6 (40)	2 (8)		7.67 (1.3–45.29)
Taking hormonal therapy				
Yes	11 (37.9)	3 (11.1)	X^2^ = 5.364 (P = 0.021)*	4.889 (1.19–20.13)
No (Ref)	18 (62.1)	24 (88.9)		1
Problems in uterus				
Yes	9 (31)	2 (7.4)	X^2^ = 4.945	5.625 (1.09–29.03)
No (Ref)	20 (69)	25 (92.6)	(P = 0.026)*	1

*Ref* reference, *X*^*2*^ Chi-square, + Monte Carlo, ^✝^Fisher, *P < 0.05.

### Estimation of SLE risk in relation to hormonal factors

The findings of the present study showed the prevalence of menstrual disorders and hormonal disturbances among SLE patients as demonstrated in Table 5. Early menopause was present in (31%) of SLE patients compared to (0%) of controls, irregular menstrual cycle was prevalent in (40%) of SLE patients compared to (8%) of controls, (37.9%) of cases used hormonal therapy compared to (11.1%) of controls, and (31%) of cases had problems in uterus versus (7.4%) of controls.

There was a statistically significant increase in SLE risk with early menopause, menstrual irregularities, use of hormonal therapy, and presence of problems in uterus (OR 31.26, 95% CI 1.7–575.54; OR 7.67, 95% CI 1.3–45.29; OR 4.889, 95% CI 1.19–20.13; OR 5.625, 95% CI 1.09–29.03 respectively).

### Estimation of SLE risk in relation to blood levels of lead, cadmium and zinc

As shown in (Table 6), the range of values of blood lead levels (Pb) in the cases group was very extensive from minimum 0.0237 mg/L to maximum 0.6951 mg/L and it is higher than the range of values in the controls group. The median concentration of blood lead in the cases group (0.115 ± 0.165) was significantly higher than in the controls group (0.067 ± 0.077). There were significantly higher blood lead levels in the cases group compared to the controls group (U = 210, P = 0.003).

**Table 6:** SLE risk estimation in relation to blood levels of lead, cadmium, and zinc(Rheumatology clinic, Alexandria University Hospital, Alexandria 2020).

Blood level	Cases (n = 29) N (%)	Controls (n = 27) N (%)	Test of significance (P value)	OR (95% CI)
Lead (mg/L)				
< 0.05 (Ref)	4 (13.8)	12 (44.4)	X^2^ = 8.847 (P = 0.012)*	1
0.05–	7 (24.1)	8 (29.6)	Chi-square trend for Odds = 7.77 (P = 0.005)*	2.63 (0.57–12)
0.09+	18 (62.1)	7 (26)		7.71 (1.85–32.21)
Minimum value	0.0237	0.0175		
Maximum value	0.6951	0.4447		
Range (maximum–minimum)	0.6714	0.4272		
Median ± IQR	0.115 ± 0.165	0.067 ± 0.077	U = 210 (P = 0.003)*	
Mean rank	34.76	21.78		
Cadmium (mg/L)				
< 0.03 (Ref)	7 (24.1)	17 (63)	X^2^ = 8.844 (P = 0.012)*	1
0.03−	11 (37.9)	4 (14.8)	Chi-square trend for Odds 4.98 (P = 0.026)*	6.68 (1.58–28.29)
0.07+	11 (37.9)	6 (22.2)		4.45 (1.18–16.8)
Minimum value	0.0068	0.0032		
Maximum value	0.3233	0.1470		
Range (maximum–minimum)	0.3165	0.1438		
Median ± IQR	0.059 ± 0.102	0.017 ± 0.042	U = 216 (P = 0.004)*	
Mean rank	34.55	22		
Zinc (mg/L)				
Minimum value	0.5412	1.1170		
Maximum value	4.7166	3.3590		
Range (maximum–minimum)	4.1754	2.2420		
Median ± IQR	2.660 ± 0.970	2.275 ± 0.707	U = 290.5, (0.098)	
Mean rank	31.98	24.76		

*Ref* reference, *X*^*2*^ chi-square, *IQR* inter quartile range, *U* non-parametric Mann Whitney test, *P < 0.05.

After categorization of the levels of blood lead in the sample, the SLE risk associated with blood lead levels ≥ 0.09 mg/L was higher and statistically significant (OR 7.71, 95% CI 1.85–32.21) in comparison with subjects having blood lead levels < 0.05 mg/L. A statistically significant trend was computed (Chi-square trend for odds =  7.77, P value = 0.005) showing that the more the increase in blood lead level, the more the chance of SLE occurrence.

The median levels of blood cadmium (Cd) in cases group (0.059 ± 0.102 mg/L) were significantly higher than in the controls group (0.017 ± 0.042 mg/L) as U = 216, P = 0.004.

As presented in (Table 6), the level of blood cadmium in the sample was categorized into groups, females having blood cadmium levels from 0.03 to less than 0.07 and ≥ 0.07 mg/L blood had 6.68 and 4.45 times more risk to develop SLE in comparison with females having blood cadmium levels < 0.03 mg/L blood and the risks for these upper two categories were statistically significant (95% CI 1.58–28.29; 1.18–16.8 respectively). The observed increased trend was found to be statistically significant (X^2^ trend for odds = 4.98, P = 0.026).

Regarding blood zinc levels (Zn), the median concentration of zinc in the controls group (2.275 ± 0.707 mg/L blood) was lower than the median concentration of zinc in the cases group (2.660 ± 0.970 mg/L blood), but the median blood zinc values in the cases and controls groups did not differ significantly (U = 290.5, P > 0.05).

**Figure 1:**
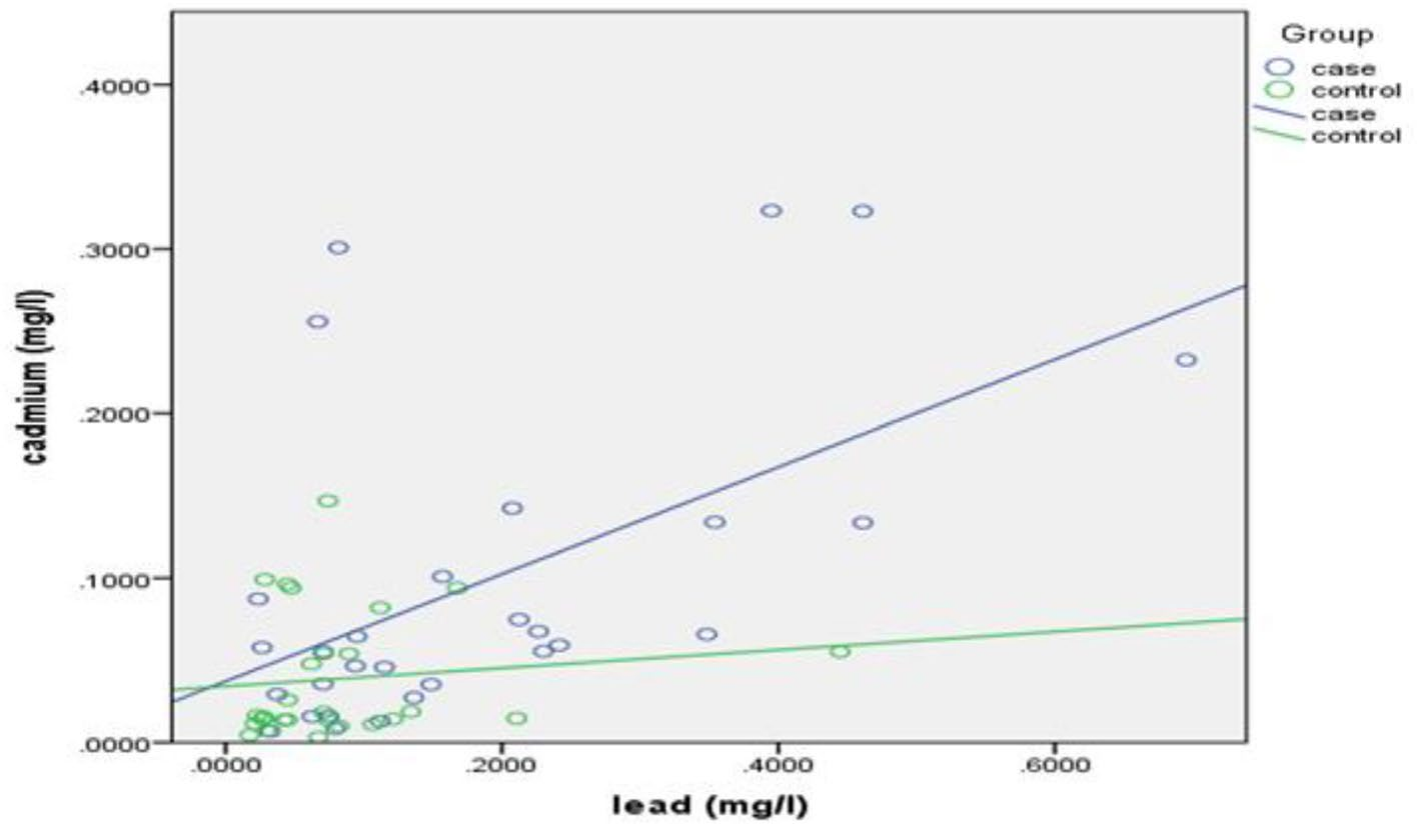
Correlation between blood lead and cadmium levels for cases and controls (Rheumatology outpatient clinic, Alexandria University Hospital, Alexandria, 2020). Performed through SPSS version 21.

Pearson’s correlation coefficient was calculated to demonstrate the relationship between lead and cadmium blood levels with each other and yielded a result of r = 0.548, P = 0.002, portraying a positive, moderate, linear, and significant correlation between lead and cadmium blood levels for the cases group (Fig. 1), whereas a non-significant correlation was observed in the controls group (r = 0.123, P = 0.542).

### Analysis of risk factors affecting SLE risk by stepwise logistic regression

A multivariate stepwise logistic regression model was built in order to determine which of these predictors really contribute to predicting SLE risk, and exclude those who do not.

Negelkerke R Square suggests that the model explains 78.4% of the variance in the outcome. The accuracy of the model was 87.5%, depicting that the model can correctly classify 87.5% of the cases. The sensitivity and specificity of the model were calculated, and they were 86.2%, and 88.9%, respectively.

As illustrated in (Table 7), the final model revealed that only five factors showed significant association with SLE. The risk was highest for subjects living near agricultural areas with an OR of 58.556 (95% CI 1.897–1807.759), followed by subjects exposed to passive smoking with an OR of 24.116 (95% CI 1.763–329.799), then subjects having blood lead levels ≥ 0.075 mg/L who were 18.981 times (95% CI 1.228–293.364) more likely to have SLE than those having blood lead levels < 0.075 mg/L, followed by subjects exposed to the sunlight who were at increased risk of SLE by 9.549 (95% CI 1.299–70.224). Whereas walking or doing exercise were significantly protective against SLE (P = 0.006); the table demonstrated a negative B coefficient (− 5.246) which indicates that a decrease in the walking and exercise is associated with a greater likelihood of SLE risk.

**Table 7:** Results of the stepwise logistic regression analysis for risk factors affecting SLE risk (Rheumatology outpatient clinic, Alexandria University Hospital, Alexandria, 2020).

Variable	Regression coefficient B	P value	Adjusted OR	95% CI	
				Lower	Upper
Living near agricultural areas (Ref1)	4.070	0.020*	58.556	1.897	1807.759
Walking or exercise (Ref2)	− 5.246	0.006*	0.005	0.000	0.220
Sun exposure (Ref3)	2.256	0.027*	9.549	1.299	70.224
Passive smoking (Ref4)	3.183	0.017*	24.116	1.763	329.799
Lead (Ref5)	2.943	0.035*	18.981	1.228	293.364
Constant	− 3.477	0.010*	0.031		

*Ref1* not living near agricultural areas, *Ref2* not walking or doing exercise, *Ref3* no sun exposure, *Ref4* no passive smoking, *Ref5* 0.075 mg/L.

Model: variables initially included: living near agricultural areas, walking and exercise, level of education, early menopause, Sun exposure, hormones, Passive smoking, and Lead, cadmium, and Zinc. pseudoR^2^_model_ = 78.4%%, which mean that the model could explain about 78% of variance in SLE outcome.

## Discussion

The current study purpose was to gain insights into the etiology of SLE and some possible risk factors.

The statistically significant difference between cases and controls regarding the education level assumes that lower education level—which is an indicator of socioeconomic status—may constitute a risk for SLE. That is similar to other studies that showed the association between lower education level and SLE onset and flare^22,23^.

The distribution of the study sample according to the residence area revealed that 48.3% of cases reported living near agricultural areas compared to 7.4% of controls, with 11.67 times more SLE risk (OR 11.67, 95% CI 2.32–58.6) compared to those not living near these areas. This may be attributed to some environmental exposures such as exposure to sunlight and pesticides used in agriculture, in addition to lower socioeconomic status, lower educational level, and poverty. This finding is in accordance with Pons-Estel et al. (2012) who concluded that rural residence was associated with high levels of disease activity at diagnosis and with renal disease occurrence in a Latin American multi-ethnic cohort (OR 1.65, 95% CI 1.06–2.57; OR 1.77, 95% CI 1.00–3.11)^24^.

Our data showed an increase of SLE risk due to sedentary lifestyle which is in accordance with a cross sectional study that showed that a high proportion of SLE patients were physically inactive with a long daily sedentary time^25^.

The statistically significant increase in SLE risk when dealing with animals or birds was in the same line with a case control study in Southern Sweden that reported a statistically significant difference between cases and controls regarding close contact with sheep^26^. This was not in the same line with the results of a recent study which supported the idea that exposures related to childhood farm residence and livestock farming may decrease susceptibility to developing SLE and that contact with livestock may confer protection against SLE^27^.

Current study findings support the role of sun exposure as a trigger for SLE, adding evidence to experimental and human studies that have shown that it can trigger disease onset and induce disease flares in SLE patients^26,28,29^.

ETS as a risk factor for SLE was not enormously previously discussed except for a cross sectional study of Brazilian SLE patients that assessed the association between smoking and SLE and confirmed that never smokers confer a 22% relative SLE risk reduction compared to ever smokers (including second hand smokers)^30^.

In addition, data from a cohort of SLE patients and controls suggested that secondhand smoke during childhood may be a risk factor for SLE (OR 1.81, 95% CI 1.13–2.89)^31^. Whereas, a US prospective cohort study concluded that Early-life exposure to cigarette smoke due to mothers’ or fathers’ smoking did not increase the risk of adult-onset SLE (RR 0.9, 95% CI 0.6–1.4; RR 1.0, 95% CI 0.8–1.3 respectively)^32^.

Our data revealed that the highest proportion of the study patients (96.6%) had negative family history of SLE. This supports the great contribution of other risk factors rather than the genetic factors in the development of SLE; suggesting the influence of environmental triggers on disease expression^8,17^. This finding was in line with a study that reported that autoimmune diseases in family members have not been associated with SLE^33^. On the other hand, a previous case control study reported the prevalence of auto-immune disease in first degree relatives in cases more than controls (53% vs 39%) and stated that a family history of any auto-immune disease was associated with increased risk of SLE (OR 6.8, 95% CI 1.4–32)^26^. This finding is also in contrast with other previous studies that stated that family history of an auto immune disease in first degree relatives (parents or siblings) was associated with increased SLE risk^34,35^.

The results of the current study regarding SLE risk in relation to hormonal factors was in concordance with a cohort study that revealed that menstrual irregularity was associated with an increased SLE^36^. In the same line comes a cross sectional study of 61 SLE patients, in which 49.2% of the patients had menstrual irregularities, of which 60% had sustained amenorrhoea (premature menopause) compared to the control group (16.7%)^37^. Moreover, a cross sectional study (N = 87) showed menstrual alterations in 37.9% of SLE patients and amenorrhea in 11.5% of patients which was higher than the general population^38^. Additionally, an increased SLE risk was reported in the NHS (Nurses’ Health Study) with the use of estrogen replacement therapy^39^. Moreover, a population‐based nested case control study found that the current use of COCs (Combined Oral Contraceptives) was associated with an increased SLE risk (RR 1.54, 95% CI 1.15–2.07)^40^.

Other studies contradicted the findings of the current study and stated that the use of HRT (Hormone Replacement Therapy) in postmenopausal SLE women did not appear to increase the rate of lupus flares and appeared to be well tolerated and safe in postmenopausal SLE patients^41,42^.

Data from experimental studies suggested that heavy metals may enhance systemic autoimmunity or accelerate disease progression in experimental models of lupus and that co-exposure to certain heavy metals may increase the risk associated with other exposures^5^.

Detailed studies on SLE onset and flares with reference to lead, cadmium, and zinc are scanty. Therefore, the current study was conducted to evaluate the role of lead, cadmium, and zinc in SLE. All blood samples were found to have lead, cadmium, and zinc concentrations.

It was observed that the median blood lead levels in the cases group as well as the controls group in this study were higher than the CDC permissible range of less than 5 µg/dL of lead in children and adults (0.05 mg/L) and this is indicative of the extent of environmental lead pollution^43^. It was also noticed that the median blood cadmium levels for both cases and controls in this study were higher than the WHO permissible range of 0.03–0.12 µg/dL of Cd^44^. This suggests more protection measures to be taken into consideration in order to avoid the toxic effects of lead and cadmium.

It was observed that the median blood zinc levels were not consistent with reference ranges of zinc in blood (70–120 µg/dL)^45^, requiring further consideration of zinc levels to avoid overdose and toxicity.

In contrast to the current study is a recent similar case control study that reported that SLE diagnosis was associated with lower serum Zn (P = 0.003), and Pb (P = 0.020)^46^. Other studies reported similar results^47,48^. However, some studies did not observe a significant difference in serum Zn concentrations between SLE patients and healthy controls which is similar to the finding of the present study^49,50^.

A case control study reported a positive correlation between lead and cadmium blood levels for the exposed group (ρ = 0.39, P = 0.023) which was consistent with the finding of the current study, whereas blood zinc levels correlated negatively with both lead (ρ = − 0.41, P = 0.015) and cadmium blood levels (ρ = − 0.44, P = 0.009). In addition, no correlations between the studied metals were found in the control group and the study suggested that zinc insufficiency is more likely to occur in cases of combined exposure to cadmium and lead, because of competition between similar ions for receptors involved in absorption, transport, storage or function, and this would explain the negative correlation in the controls group between zinc blood levels with either lead and cadmium levels^51^.

### Limitations of the study

- The potential of case control studies for recall bias and misclassification error; as most exposure information were based on self-reported history so some inaccuracies can be expected. The design of the study also limited the ability to ascribe causal relationships to the associations detected and to control for all potential confounders.
- Absence of genetic information for participants, so the potential effects of genetic heterogeneity on the association between risk factors and SLE risk could not be determined.
- Human population is rarely exposed to a single agent over time, and there may be a significant delay between exposure and the onset of the disease.
- The small sample size may make it difficult to determine if a particular outcome is a true finding and in some cases no difference between the study groups is reported.

## Conclusion and recommendations

To date, our knowledge about the etiology of SLE is still unclear and limited; genetic and environmental interactions were suggested.

From the present case control study, it is concluded that the risk portion attributed to unsafe and unhealthy environment was found to be quite significant, showing how the environment can play an important role in SLE occurrence.

Exposure to potential environmental risk factors specifically heavy metals should not be under-estimated. Hence, there is an urgent need for interventions to reduce environmental risk factors exposure in order to achieve substantial public health gains.

Increasing the awareness of patients about the environmental pollutants and the ways to protect their health is necessary. As well as the awareness of health care providers about environmental risk factors, so they can advise the patients about the ways of reduction of exposure to environmental hazards. Educational awareness programs to patients and their family should be carried out through media and non-governmental organizations (NGOs) to raise their knowledge about environmental hazards and how to minimize the sources of their exposure and their consequent negative impacts.

Additional experimental and epidemiological studies are required to determine the causative role of several environmental exposures, to confirm data from case control studies.

## Methods

This case control study was conducted at the Rheumatology outpatient clinic of Alexandria Main University Hospital. The Inclusion criteria were female patients diagnosed with idiopathic SLE according to SLICC criteria^52^. The controls included healthy females who accompanied the SLE patients who came from remote rural areas in their visit to the Rheumatology outpatient clinic. The cases and controls were matched for age and parity.

Exclusion criteria included (1) male SLE patients “they were excluded mainly to avoid gender bias that may affect the results due to the hormonal effect. Besides, the disease affects mainly females”; (2) drug-induced Lupus; (3) overlap syndrome as lupus and rheumatoid arthritis; (4) any other rheumatic diseases; (5) coexisting morbidity not related to SLE, e.g. diabetes, hypertension; (6) cancer; (7) dementia or psychosis; (8) intake of nutritional supplements in the 6 months prior to the blood collection.

### Sample size

Based on a previous case control study, the mean of serum Zinc among systemic lupus erythematosus (SLE) was 700.61 ± 135.91 and among controls was 860.45 ± 123.74, using an alpha error of 0.05 and power 98%^48^. The minimum required sample size was estimated to be 46 adults, 23 for each group, which was increased to 56 adults, 29 cases and 27 controls. The sample size was calculated using G. Power software.

### Data collection methods and tools

1. A pre-designed pre-coded structured interviewing questionnaire was used to collect data from all participants (cases and controls). It included personal and socio-demographic data, data about occupation, lifestyle factors, the medical history, the smoking history including exposure to passive smoking, and some possible indoor environmental risk factors aiming to ascertain exposure to some environmental risk factors suspected to affect SLE risk. Regarding the evaluation of poor water quality, the participants were asked about the availability and quality of drinking water, any problems concerning drinking water (clarity, taste, smell), the type of drinking water pipes (with or without lead) by asking them whether they are old or newly installed, and the drinking water source (filtered, bottled, or tap water). Whereas for sun exposure, they were asked about the daily exposure to the sun, duration of exposure, wearing of protective clothes, and use of sunscreen, as well as whether there is an occupational sun exposure. Concerning the evaluation of sedentary lifestyle, they were questioned whether they perform any physical activity and the duration per week, whether they prefer walking or taking the car, and the duration of watching TV.

Questions of the questionnaire were taken from similar previously validated Arabic and English research questionnaires^26,53^. In addition, it was assessed by an expert at the faculty of medicine, Alexandria University. The English version of the questionnaire underwent a forward and back translation by native speakers whom are experts in public health.

2. Laboratory investigation

A blood sample (5 mL) was drawn from each participant to measure blood levels of lead, cadmium, and zinc. The samples were transferred into heparinized collection tubes. Cadmium, lead, and zinc were extracted from the blood samples using the conventional wet acid digestion method using concentrated nitric acid (HNO_3_). A blank using deionized water instead of blood was done for each batch of analysis for comparison^44,54^. The digested samples were filtered and were subjected to elemental analysis using flame atomic absorption spectrophotometer (Shimadzu model AA-6650) at the central laboratory of the High Institute of Public Health (HIPH), Alexandria University.

### Statistical design

SPSS version 21 was used for data entry and analysis. Qualitative variables were described through number and percentages of cases. For quantitative continuous variables, tests of normality were done. Mean and standard deviation (mean ± SD) were calculated if the variable follows normal distribution, and median and interquartile range (median ± IQR) if it does not follow normal distribution. “Pearson’s Chi-square test (X^2^)” was used to calculate significant differences between cases and controls for the categorical data. If the assumptions were violated, “Fisher’s exact test” (if 2*2 table) or “Monte Carlo test” (if m*n table) were used.

Differences between the means of the two groups were examined using independent t test for the continuous, normally distributed variables. The Mann–Whitney–Wilcoxon non-parametric test (U), for the continuous, non-normally distributed variables. Odds ratio (OR) was calculated to measure SLE risk. Increasing trends in SLE risk concerning some risk factors were tested using “Chi-square for trend”. Pearson’s correlation coefficient was used to test the association between quantitative variables.

A multivariate stepwise logistic regression analysis was used to see if there were significant associations between specific exposures and SLE and to adjust for some potential confounders. Negelkerke R^2^ was calculated to tell the amount of variation in SLE risk which is explained by the model.

### Ethical approval

Approval of the Ethics Committee of the High Institute of Public Health for conducting the research was obtained. Approval for conducting the study at the Rheumatology Outpatient Clinic of Alexandria Main University Hospital was obtained from the hospital outpatient clinics director after a formal written request for that. The study was performed in accordance with the ethical standards in the Declaration of Helsinki. A written informed consent was taken from all study participants after explanation of the purpose and benefits of the research. Anonymity and confidentiality were ensured. All methods were performed in accordance with the relevant guidelines and regulations.

## Data Availability

The datasets used and/or analyzed during the current study are not publicly due to privacy and ethical concerns but are available from the corresponding author on reasonable request.
